# Development of RNAi Methods for *Peregrinus maidis*, the Corn Planthopper

**DOI:** 10.1371/journal.pone.0070243

**Published:** 2013-08-07

**Authors:** Jianxiu Yao, Dorith Rotenberg, Alireza Afsharifar, Karen Barandoc-Alviar, Anna E. Whitfield

**Affiliations:** 1 Department of Plant Pathology, Kansas State University, Manhattan, Kansas, United States of America; 2 Plant Virology Research Center, College of Agriculture, Shiraz University, Shiraz, Iran; University of Tennessee, United States of America

## Abstract

The corn planthopper, *Peregrinus maidis,* is a major pest of agronomically-important crops. *Peregrinus maidis* has a large geographical distribution and transmits *Maize mosaic rhabdovirus* (MMV) and *Maize stripe tenuivirus* (MSpV). The objective of this study was to develop effective RNAi methods for *P. maidis*. Vacuolar-ATPase (V-ATPase) is an essential enzyme for hydrolysis of ATP and for transport of protons out of cells thereby maintaining membrane ion balance, and it has been demonstrated to be an efficacious target for RNAi in other insects. In this study, two genes encoding subunits of *P. maidis* V-ATPase (*V-ATPase B* and *V-ATPase D*) were chosen as RNAi target genes. The open reading frames of *V-ATPase B* and *D* were generated and used for constructing dsRNA fragments. Experiments were conducted using oral delivery and microinjection of *V-ATPase B* and *V-ATPase D* dsRNA to investigate the effectiveness of RNAi in *P. maidis*. Real-time quantitative reverse transcriptase-PCR (qRT-PCR) analysis indicated that microinjection of *V-ATPase* dsRNA led to a minimum reduction of 27-fold in the normalized abundance of *V-ATPase* transcripts two days post injection, while ingestion of dsRNA resulted in a two-fold reduction after six days of feeding. While both methods of dsRNA delivery resulted in knockdown of target transcripts, the injection method was more rapid and effective. The reduction in *V-ATPase* transcript abundance resulted in observable phenotypes. Specifically, the development of nymphs injected with 200 ng of either *V-ATPase B* or *D* dsRNA was impaired, resulting in higher mortality and lower fecundity than control insects injected with *GFP* dsRNA. Microscopic examination of these insects revealed that female reproductive organs did not develop normally. The successful development of RNAi in *P. maidis* to target specific genes will enable the development of new insect control strategies and functional analysis of vital genes and genes associated with interactions between *P. maidis* and MMV.

## Introduction

The corn planthopper, *Peregrinus maidis* (Hemiptera: Delphacidae), is a widely distributed and destructive insect that causes significant yield losses by feeding on important crops such as corn, sorghum, and pearl millet. The insect has three main developmental stages: eggs, 1^st^–5^th^-stage nymphs and adults. On average, the female oviposits 17±2 eggs per day and can produce more than 300 eggs during its adult life span [1]. Corn planthoppers develop into one of three winged adult forms: macropters (long-winged), koeliopters (short-winged), and brachypters (wingless), depending on the type of habitat and population density [2]. Higher population density of nymphs promotes the appearance of macropters, and the long-distance migration of macropters allows *P. maidis* to exploit temporary crop or weed habitats, where they are capable of surviving on multiple hosts [3]. A persistent habitat with favorable and stable environmental conditions favors brachypters [2]. The male and female macropters and female brachypters are common, while koeliopters occur in lower numbers in the field [3].

*Peregrinus maidis* causes direct damage by feeding on vascular tissues via piercing-sucking mouthparts. Vascular feeding on the leaf midrib, whorl, and sheath causes stunting, leaf chlorosis and reduced plant vigor. Severe infestations result in basipetal withering of leaves and even death of the plant [4]. Feeding and punctures caused by oviposition also lead to leaf desiccation and reddening [3]. The insect damages the plant indirectly by producing copious amounts of honeydew that is excreted on plant surfaces and provides a substrate for sooty mold growth, which in turn impairs photosynthetic capacity of the leaves [5].

The most significant indirect damage caused by *P. maidis* is virus transmission. *P. maidis* is the only known vector of *Maize mosaic rhabdovirus* (MMV) and *Maize stripe tenuivirus* (MSpV) [6], [7]. MMV infection causes yellow spots or interrupted stripes, bands between and along the fine veins, and plants can be severely stunted. The typical symptoms on plants appear from four days to seven weeks after viruliferous-*P. maidis* infestation. The combination of damage caused by *P. maidis* feeding and virus infection of the plant has the potential to significantly reduce crop yields [8], [9].

MMV is transmitted by *P. maidis* in a persistent propagative manner, *i.e.*, the virus replicates in the vector. The virus persists in the vector for the duration of its life and the insect is capable of transmission after a latent period of approximately seven days [10]. MMV infection of *P. maidis* begins in the midgut and spreads to the esophagus, compound eyes, nerve ganglia, visceral muscle, hemocytes, tracheae, salivary glands and other tissues [11]. Due to the propagative nature of the virus, infected planthoppers have the potential to transmit virus for an extended period of time and one insect could effectively inoculate many individual plants.

The most common management strategies used for control of *P. maidis* include chemical treatments and host resistance. Insecticides such as endosulfan and carbaryl have been used for management of *P. maidis* on corn. Although insecticide control of *P. maidis* has been a convenient and effective option in the past, indiscriminate usage has resulted in resistance leading to a resurgence of the insect, and has led to serious environmental pollution [12]. Hence, plant host resistance is a preferred alternative control strategy. However, few sources of genetic resistance have been reported for corn, sorghum, or millet [3].

Plant-mediated RNA interference (RNAi) has been proposed as a promising approach for controlling insect pests [13]. RNAi is a highly specific mechanism that functions to inhibit the expression of a gene at the post-transcriptional level via degradation of its RNA transcript. It is also an effective technique to study gene function by the observable phenotype of an organism whose target gene is silenced. Long dsRNAs (300–500 bp) are commonly employed in insect molecular biology studies. Double-stranded RNA (dsRNA) that targets the gene transcript encoding vacuolar ATP synthase subunit A (*V-ATPase A*) has been shown to kill *Diabrotica virgifera virgifera* larvae in feeding assays [14]. Double-stranded RNA ingestion and injection have also been used to reduce target gene transcript levels in the brown planthopper, *Nilaparvata lugens* [15], [16]. Chen et al. [15] found that *N. lugens* ingestion of trehalose phosphate synthase dsRNA led to the reduction of target gene transcripts, subsequent decrease in the activity of the enzyme, and a significant increase in mortality. Thus, ingested dsRNA can be insecticidal and careful design of dsRNA constructs can result in species-specific insecticides [17], [18]. Crops specifically designed to express dsRNAs have the ability to target and suppress vital gene transcripts in specific pests upon ingestion, like P450 monooxygenase [18] and subunits of V-ATPase [14], resulting in control of insect pests.

One of the most attractive candidates for RNAi via ingestion and injection is V-ATPase, an evolutionarily conserved enzyme. In general, the V-ATPase pump is localized in the apical membranes of nearly all epithelial tissues of insects, such as those found in Malpighian tubules, salivary glands, labial glands, sensory sensilla, and the midgut [19]. The V-ATPase pump plays an essential role in nutrient uptake and ion balance in the insect gut system [20]. The V-ATPase holoenzyme functions by hydrolyzing an ATP molecule to ADP and phosphate and using this generated energy to pump protons across plasma membranes to regulate pH in various intracellular compartments [21]. V-ATPase is composed of two functional domains, V1 and V0. The V1 domain, located on the cytoplasmic side of the membrane, is comprised of eight different subunits (A–H) and is responsible for ATP hydrolysis. The V0 domain is the membrane-bound protein, composed of five subunits (a – e) and functions in proton-conducting [22]. *Drosophila melanogaster* mutants of the V-ATPase B subunit (V0 domain) have a lethal phenotype and the Malpighian tubules appear transparent due to the accumulation of uric acid crystals [23]; and knockdown of subunit B of V-ATPase killed all tested flies [24].

In this study, we used RNAi to specifically target mRNAs essential for V-ATPase activity. The goals of the project were to compare the efficacy of two dsRNA delivery methods and determine tljkhe phenotype associated with silencing *V-ATPase B* and *D* subunits in *P. maidis*. The synthesized dsRNA was delivered via ingestion and injection and we observed a significant reduction in transcript abundance using both delivery methods. This study established the standard methods of exogenous dsRNA delivery in *P. maidis* and documented the essential function of V-ATPase in female oogenesis in this insect. *P. maidis*-MMV has become an important model system for investigation of insect-rhabdovirus interactions and a high quality gut-specific cDNA library [25] provided the *P. maidis* sequences for functional studies using RNAi-based strategies. RNAi will be an important tool to study protein function in *P. maidis* and the molecular mechanisms underpinning vector competence.

## Materials and Methods

### Insect rearing

Corn planthoppers were reared on young corn plants with four to six leaves (cultivar Early Sunglow, Syngenta Inc., Greensboro, NC) and kept in insect-proof 12 in×12 in×24 in cages covered by nylon-mesh (46-micron) screen (customized insect-proof cage, Bioquip Inc, Compton, CA) in a plant growth chamber at a temperature of 24±1°C and a photoperiod of 14-h light: 10-h dark. In order to synchronize insect developmental stages for dsRNA exposure, mature adults were placed on fresh corn plants for a 24 h-oviposition period, and the adults were removed from plants the following day. Emergent nymphs were moved to a new cage with fresh corn plants after each molt and developmental stage was confirmed by microscopic examination.

### cDNA sequencing of *RPL10*, *V-ATPase D* and *B* subunit genes

Partial cDNA sequences of *RPL10 (ribosomal protein L10)*, *V-ATPase B* and *V-ATPase D* were identified from the *P. maidis* gut- specific EST library using Blast2GO [25]. The EST library contained two full-length and one partial open reading frame (ORF) for *RPL10*, *V-ATPase D* and *V-ATPase B*, respectively. To obtain a full-length ORF sequence for *V-ATPase B,* 5` Rapid Amplification of cDNA Ends (RACE) (Clontech SMART^TM^ RACE cDNA amplification kit, Mountain View, CA) was performed on 1 µg of total RNA extracted from a group of five 5^th^ instar nymphs using the Qiagen RNeasy mini kit (Valencia, CA). Touchdown PCR of the 5`RACE product was performed with one *V-ATPase B* gene-specific primer (5`-CACATCACTGTGGTCCTTACGGGTCAT-3`) designed using Primer 3 software and a universal primer (5`-CTAATACGACTCACTATAGGGCAAGCAGTGGTATCAACGCAGAGT–3′) provided by Clontech RACE cDNA kit. The resulting PCR products were sub-cloned using the Invitrogen TA-vector system (Invitrogen Inc. Carlsbad, CA) and sequenced using the ABI 3700 DNA sequencer at the KSU DNA Sequencing Facility (Manhattan, KS).

### dsRNA synthesis

One microgram of *P maidis* total RNA (as described above) was used for cDNA synthesis using Thermo Scientific Verso cDNA Synthesis kit (Waltham, MA). Using 1.0 µL of the cDNA sample as template, the target sequence fragments were amplified by RT-PCR using template-specific primers (Table 1) conjugated with the 23 bp T7 RNA polymerase promoter. GFP fragments were amplified from the pSITE-2NB vector [26]. One microgram of each PCR product (447 bp for *V-ATPase D*, 420 bp for *V-ATPase B* and 305 bp for *GFP*) was used as template for dsRNA synthesis using the T7 Ribomax^TM^ Express RNAi System (Promega, Madison, WI). The dsRNA was ethanol precipitated overnight, re-suspended in RNase-free water, and quantitated at 260 nm using a NanoDrop 2000 Spectrophotometer (Thermo Fisher Scientific Inc. Carlsbad, CA). The quality and integrity of dsRNA were determined by agarose gel electrophoresis.

**Table 1:** Primer sequences used for dsRNA synthesis and real-time quantitative reverse transcriptase-PCR (qRT-PCR).

Gene	Primer use	Sequence (5′–3′)	Product length	Primer efficiency
GFP	dsRNA synthesis	T7F: GGATCCTAATACGACTCACTATAGGGTGACCACCCTGACCTAC	305bp	
		F: GTGACCACCCTGACCTAC		
		T7R: GGATCCTAATACGACTCACTATAGGTTGATGCCGTTCTTCTGC		
		R: TTGATGCCGTTCTTCTGC		
				
*V-ATPase D*	dsRNA synthesis	T7F: TAATACGACTCACTATAGGGATCCAGAGGAGCCCAAACTT	447bp	
		F: ATCCAGAGGAGCCCAAACTT		
		T7R: TAATACGACTCACTATAGGGTCGTCCAAAGTGACGAAAGA		
		R: TCGTCCAAAGTGACGAAAGA		
				
*V-ATPase B*	dsRNA synthesis	T7F: TAATACGACTCACTATAGGGAACGGCTCCATCACTCAGAT	420bp	
		F: AACGGCTCCATCACTCAGAT		
		T7R: TAATACGACTCACTATAGGGCAGCAGCTGCCAACCAAT		
		R: CAGCAGCTGCCAACCAAT		
				
*V-ATPase D*	qRT-PCR	F: CGTTATCATACCTAGAATTG	199bp	111%
		R: TTCATCCAGCATATTGTT		
				
*V-ATPase B*	qRT-PCR	F: AAATTCCCATCTTCTCTG	128bp	112%
		R: AATAGCAAAGTTGTCCTC		
				
*RPL10*	qRT-PCR	F: CGAAGAAATGGGGTTTCA	105bp	115%
		R: ACTCGCCTTGTATCTGTCG		

### dsRNA delivery by ingestion

A time-course experiment was conducted to determine the efficiency of gene silencing by feeding planthoppers dsRNA. The experiment design consisted of two treatments, dsRNA of *V-ATPase B* or *V-ATPase D* and dsRNA of *GFP* (negative control), and three experimental replicates per treatment in a complete randomized design. The experiment was performed once. Groups of 45 third-stage nymphs were collected and anesthetized with carbon dioxide gas for 30 sec, then each group was placed into a feeding chamber constructed from a 3 cm (diameter) ×5 cm (length) plastic cylindrical tube (BD Bioscience, Durham, NC) with one end covered by insect-proof nylon mesh (48-micron) screen (Small Part Inc. Seattle, WA) and the other end covered by two-layers of Parafilm sandwiched together. Twenty-microliters of liquid diet, consisting of 10% sucrose, 60% GIBCO SF-900II^TM^ serum free medium (Invitrogen Inc. Carlsbad, CA), and 0.5 µg/µL dsRNA was placed between the Parafilm layers and replaced daily. Three live insects were collected and pooled per treatment (n = 3 independent replicates per treatment) after 2-, 4-, and 6-days exposure to the feeding solution. The insects were homogenized in 200 µL Trizol reagent (Invitrogen, Carlsbad, CA, USA) and stored at −80°C until processed for total RNA extraction and real-time RT-PCR analysis of gene silencing.

### dsRNA delivery by injection

A similar time-course experiment as described above was conducted to determine the efficiency gene silencing after dsRNA-injection. The experiment included three treatments (dsRNA of *V-ATPase B, V-ATPase D,* and *GFP*) and three experimental replicates per treatment in a complete randomized design. The experiment was performed twice; one experiment tested adults and the other tested nymphs. Sixty nymphs or adults were collected and CO_2_ – anesthetized in 1.5 cm (diameter) ×15 cm (length) plastic cylindrical tubes (BD Bioscience, Durham, NC) with a 0.5 cm (diameter) opening at the bottom of tube for delivery of CO_2_ gas. The anesthetized insects were placed in a petri dish that was stored in a bucket of ice for continued immobilization of the insects. The immobilized insects were then placed in an injection arena (10-cm-diameter petri dish containing a layer of 1% agarose) and injected immediately. The connective membrane between the third and fourth dorsal abdominal segments of each insect was pierced once to deliver 50 nL of dsRNA (250 ng) of each treatment at 10 nL/sec using a Nanoinjector II (Drummond Scientific, Broomall, PA). The injected adults or nymphs were placed on a healthy corn plant to recover and reared at 24±1°C and a 14/10 h light/dark photoperiod in a ten-corn seedlings pot covered with a 3 in (diameter) ×12 in (length) clear plastic tube (Uline Inc. WI, USA) capped with an insect-proof screen. Three live insects were collected and pooled per replicate (n = 3 independent replicates per treatment) at two day intervals post injection. Insects injected as nymphs were collected 2 to 12 days after injection and adults 2 to 6 days after injection. The insects were homogenized in 200 µL Trizol reagent and stored at −80°C until processed for real-time RT-PCR analysis.

### The effect of RNAi of *V-ATPase B* or *D* on nymph survival after dsRNA treatment by feeding or injection

Separate time-course experiments were conducted to determine the effect of dsRNA feeding or injection on nymph mortality using the same experimental design, treatments, and parameters as described above with a few exceptions. For the dsRNA feeding experiment, treatment replication was achieved over three independent experiments (*i.e.*, three biological replicates per treatment). At each time point, one chamber per treatment was removed from the experiment and the number of live and dead nymphs was recorded at two, four and six days of feeding. For the dsRNA injection experiments, the experiment was performed twice (two independent biological replicates) with three experimental replicates per treatment, and a water control was also included. In each experiment, around 60 5^th^ nymphs were injected with 40 nL (200 ng) of dsRNA (*V-ATPase B*, *V-ATPase D* and *GFP*) or H_2_O, respectively. The injected nymphs (∼60 insects) of each treatment were equally divided into three small clear cages with dimensions of 1.5 in (diameter) ×12 in (length) (Uline Inc. WI, USA), and each cage enclosed around 20 individual nymphs. Every three days, the number of dead and live insects was recorded up to 18 days post injection.

### Impact of RNAi of V-ATPase on female fecundity

During the mortality experiments, we observed that *V-ATPase D* or *V-ATPase B* dsRNA-injected insects appeared to oviposit fewer eggs into corn plant tissues compared to those injected with *GFP* dsRNA or H_2_O. Thus, experiments were conducted to determine the effect of dsRNA-knockdown of *V-ATPase B* or *V-ATPase D* on reproductive capacity and organ/oocyte morphology of *P. maidis*. Young adults were the subjects of experiments to document fecundity, *i.e.*, number of eggs oviposited in plant tissue and reproductive organ/oocyte morphology in dissected females, and 5^th^-stage nymphs were subjects of a second trial to document morphology only. Each experiment was performed twice. In the adult experiment, newly emerged, 24-h-old females and males (n = 20 of each sex) were collected and injected with 50 nL (250 ng) of dsRNA of *GFP*, *V-ATPase B* and *V-ATPase D,* respectively. Following injection, five replicate mating/oviposition cages (1.5 in diameter ×12 in length) were prepared per treatment with each cage containing two females and two males on a single 7-day-old healthy corn plant. Seven days after injection, eggs laid in corn leaves were visualized readily and enumerated with the aid of a stereo-microscope. The surviving females were collected from each treatment cage and frozen at −80°C for dissection to expose ovaries and oocytes. In the nymph trial, each dsRNA treatment consisted of three replicate cages containing 20 5^th^ instar nymphs injected with 40 nl (200 ng) dsRNA. Seven days after adults emerged, all females were collected and dissected in the same manner as described above.

### Real-time quantitative RT-PCR

**Figure 1:**
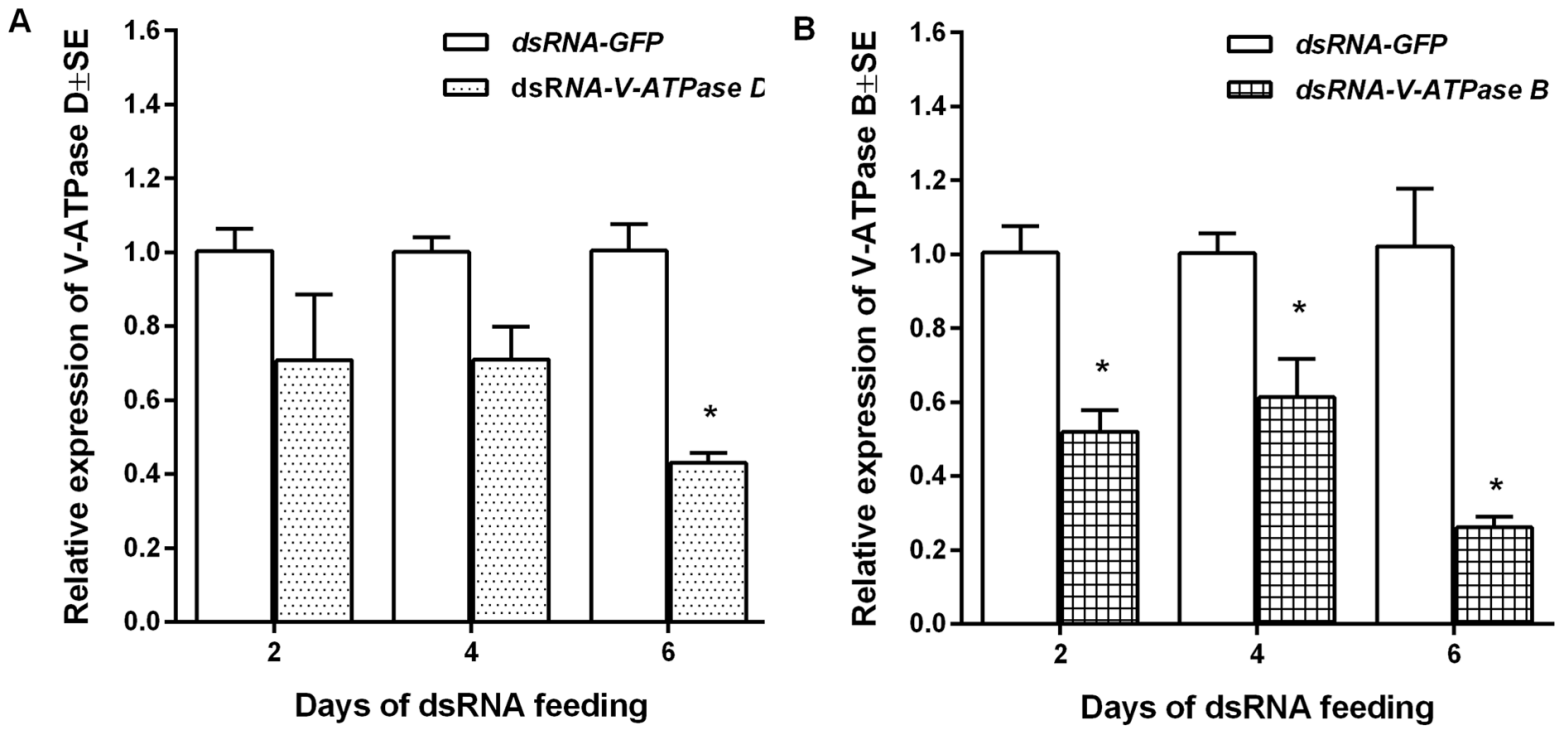
Reduction in the normalized abundance of *V-ATPase D* and *V-ATPase B* transcripts in *Peregrinus maidis* by oral delivery of dsRNA. Third instar planthoppers were allowed to feed on dsRNA of *V-ATPase D,* dsRNA of *V-ATPase B* or dsRNA of GFP as a negative control treatment for up to six days. Insects were harvested two, four, and six days after feeding on dsRNA for real-time quantitative reverse transcriptase-PCR analysis of gene knockdown. The abundance of *V-ATPase D* (**A**) and *V-ATPase B* (**B**) transcripts was normalized to *P. maidis* ribosomal L10 transcripts. Each bar represents the mean and standard error of the mean of n  =  three experimental replicates each consisting of a group of three insects. The asterisk (*) indicates that treatment means differ significantly (*P*<0.05) at a given time-point.

**Figure 2:**
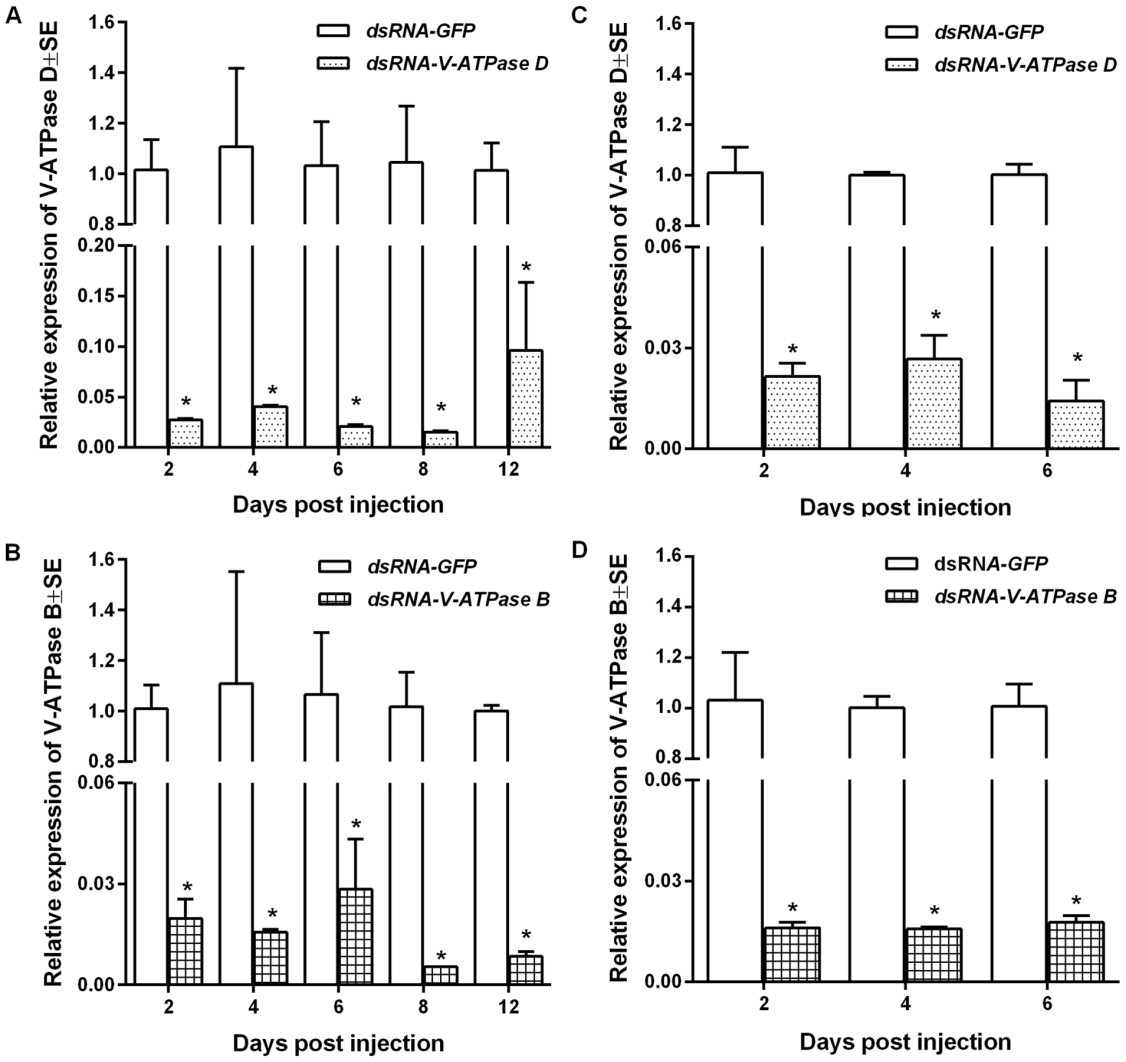
Reduction in the normalized abundance of *V-ATPase B* and *D* transcripts in *Peregrinus maidis* by injection of dsRNA. (**A and B**) 5^th^-stage nymphs or (**C and D**) young adults of *P. maidis* were injected with 50 nL of dsRNA (250 ng) of *V-ATPase D*, *V-ATPase B*, or *GFP* as a negative control and harvested every two days for real-time quantitative reverse transcriptase-PCR analysis of gene knockdown. The abundance of *ATPase B* and *V-ATPase D* transcripts was normalized to *P. maidis* ribosomal L10 transcripts. Each bar represents the mean and standard error of the mean of n  =  three experimental replicates each consisting of a group of three insects. The asterisk (*) indicates that treatment means differ significantly (*P*<0.05) at a given time-point.

We performed real-time quantitative reverse transcriptase-PCR (qRT-PCR) to determine the normalized abundance of target transcript (*V-ATPase D* or *B*) to an internal reference transcript (*RPL10*), a stably-expressed gene identified in our *P. maidis* EST library (GenBank dbEST accession: GO438398), for insects given dsRNA orally or by injection. Expression stability of actin, β-tubulin, and ribosomal proteins RPI8 and RPL10 were compared using BestKeeper, and RPL10 was found to be the best internal reference gene (K. Barandoc-Alviar, unpublished). Total RNA was extracted from each pool of insects using Trizol and RNA quantity analyzed by NanoDrop 2000 Spectrophotometer (Thermo Fisher Scientific Inc. USA) at 260 nm. The first strand cDNA of each pool was synthesized from 1 µg total RNA using the ThermoScientific Verso cDNA synthesis kit with RT-enhancer (ThermoScientific Inc. Waltham, MA) to remove residual DNA contamination. Gene-specific primer pairs were designed using Beacon7 software (Table 1) and 9 µL of cDNA (diluted 1:50) was used as template in a 20-µL SYBR Green Supermix reaction (Bio-Rad Inc. Hercules, CA). Quantitative PCR was performed in a 2-step amplification with 40 cycles of 95°C for 30 s and 56°C for 30 s using a Bio-Rad IQ thermocycler (Bio-Rad Laboratories Inc. Hercules, CA). The normalized abundance of target RNA (V-ATPase D or B) to the internal reference RNA (RPL10) was calculated (ΔCT) for the treatment (dsRNA-ATPase) and the control (dsRNA-GFP) samples. The relative abundance of target RNA (V-ATPase D or B) in the treatment (dsRNA-ATPase) compared to the control (dsRNA-GFP) was calculated using the 2-ΔΔCT relative expression method [27]. For this calculation, the normalized target RNA value for each treatment sample was divided by the average normalized values for the control samples. For comparative illustration (Figures 1 and 2), the relative expression of V-ATPase D or B in each of dsRNA-GFP control samples was calculated in the same manner.

### Statistical analyses

All statistical analyses were performed using SAS v9.2 (SAS Institute, Cary, NC) to determine if there were significant effects of the dsRNA*-V-ATPase* treatment on i) normalized abundance of target gene transcripts (*V-ATPase D* and *B*) in adults and nymphs over time; ii) nymph survival (proportion of live insects) over time; and iii) fecundity (number of oviposited eggs). Prior to analysis, normalized abundance values were log_10_-transformed to normalize data and associated residuals. Analysis of variance was performed as a factorial design with the statistical model consisting of two main effects (dsRNA-treatment and time) and the interaction term (dsRNA treatment*time). The model was tested using the SAS procedure PROC MIXED and the LSMEANS statement was used to generate least square differences and p-values for making comparisons between treatments over time, and between two independent biological replicates. The survival data (proportion of live insects) were arcsine square root-transformed prior to analysis. For the feeding experiment, analysis of variance of the survival data was performed using the same model and SAS procedure as above. For the injection experiment, because the same cohort of insects in a given experimental replicate cage was monitored repeatedly over time, the PROC MIXED procedure include the REPEATED statement, with cages of insects as subjects measured over time, and the LSMEANS statement was used accordingly.

For egg count data, a Poisson regression analysis with a log-link function (*i.e*, log-linear model) was performed using the SAS procedure GENMOD. The model consisted of one main effect (dsRNA treatment). Type I and Type III analyses were performed to test the significance of the treatment main effect, and least square differences and p-values were generated to compare treatment means.

## Results

### Sequence analysis of full-length cDNA of *RPL10*, *V-ATPase D* and *B*

In this study, we identified two cDNA sequences of *V-ATPase* subunits (*B* and *D*) and one *RPL10* (internal reference) sequence from a *P. maidis* gut-specific EST collection (Whitfield et al., 2011). Full length cDNA sequences of *RPL10* (636 nt), *V-ATPase D* (985 nt) (Table 2, GenBank accession: KC473162) were obtained from cDNA library plasmid re-sequencing. *V-ATPase B* (2495 nt) was obtained from 5` RACE and submitted to NCBI GenBank (Table 2, GenBank accession: KC473163). The predicted ORFs for *V-ATPase D* and *B* were 741 nt and 1503 nt, respectively. Blastx analysis of ORF nucleotide sequences revealed that both V-ATPase B and D subunits are well-conserved sequences having more than 90% identities in amino acid sequence between *P. maidis* and *Acyrthosiphon pisum,* another Hemipteran. For example, the *V-ATPase B* subunit sequences of these two species were 94% identical at the amino acid level and 77% identical at the nucleotide sequence level. The two *P. maidis* V-ATPase sequences shared more than 90% identity in amino acid sequence when compared to species belonging to different insect orders, such as *Manduca sexta*, *Drosophila melanogaster*, *Aedes aegpyti*, and *Tribolium castaneum*.

**Table 2:** Features of full-length cDNAs of *V-ATPase B*, *V-ATPase D* and *RPL10* from *Peregrinus maidis* and their predicted translated proteins.

Putative gene	ORF (nt)	Predicted amino acids (aa)	Accession No
*V-ATPase B*	1500	500	KC473163
*V-ATPase D*	741	247	KC473162
*RPL10*	636	212	KC473164

### *V-ATPase B* and *V-ATPase D* knockdown in dsRNA-fed nymphs

Our first attempt to knock down V-ATPase gene expression in *P. maidis* was by feeding nymphs dsRNA of the *V-ATPase D* and *V-ATPase B* subunit continuously over a period of six days with sampling at 2-day intervals. Real-time qRT-PCR analysis revealed that there were marginal to no apparent differences in the normalized abundance of endogenous *V-ATPase D* transcripts between the nymphs fed dsRNA of *V-ATPase D* compared to those fed dsRNA of *GFP* at two (*P* = 0.06) and four days of feeding (*P* = 0.11). By six days, however, there was a significant 2.3-fold reduction (*P* = 0.0014) in the normalized abundance of *V-ATPase D* transcripts in nymphs fed dsRNA of *V-ATPase D* compared to the control group (Fig. 1A). However, significant differences in the normalized abundance of *V-ATPase B* transcripts were detected in nymphs fed dsRNA of *V-ATPase B* at 2-days (*P* = 0.006), 4-days (*P* = 0.029) and 6-days (*P* = 0.0087) after feeding (Fig. 1B).

### *V-ATPase D* and *V-ATPase B* knockdown in dsRNA-injected nymphs and adults

As a means of enhancing the magnitude and timing of gene knockdown in *P. maidis*, we delivered dsRNA of the target genes directly to the hemolymph of the insect by micro-injection. In contrast to the dsRNA feeding experiments, delivery of dsRNA of *V-ATPase* subunits by injection resulted in significant reductions (*P*<0.0001) in *V-ATPase B* or *V-ATPase D* transcripts in nymphs by two and four days after injection as compared to the dsRNA of *GFP* control group (Fig. 2A and B). On average, injection of nymphs with dsRNA of *V-ATPase B* resulted in target reductions ranging from 37- to 90-fold over the course of the six-day sampling period, with no significant differences between time-points (*P*>0.4). Injection of nymphs with dsRNA of *V-ATPase D* resulted in 27- to 50-fold reductions in *V-ATPase D* transcripts on average over the same time frame, with enhanced knock-down of this gene by day 6 compared to the earlier time points (*P*<0.04). A significant knockdown (10-fold reduction) of these genes compared to the dsRNA-*GFP* control group for nymphs persisted up to 12 days post injection. The high mortality of insects injected with dsRNA of *V-ATPase* precluded reliable measurement of knockdown beyond 12-days. Comparable to the reaction of nymphs to dsRNA-*vATPase D* injection, young adults experienced 37- to 70-fold reductions (*P*<0.0001) in *V-ATPase D* and *V-ATPase B* transcripts compared to the control group over the three time-points (Fig. 2C–2D).

### Mortality of nymphs fed dsRNA of *V-ATPase B* or *D*

**Figure 3:**
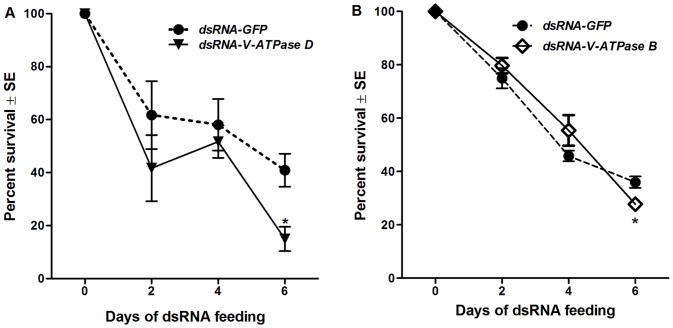
*Peregrinus maidis* mortality after ingestion of dsRNA. The proportion of live 3^rd^-stage nymphs was determined at 2-day intervals after feeding with artificial diet containing 500 ng/uL dsRNA of *V-ATPase D*, dsRNA of *V-ATPase B* or dsRNA of *GFP* as a negative control for up to six days. Each point represents the mean ± the standard error of the mean of n  =  three biological replicates, *i.e.*, independent trials, with groups of 30 insects permanently removed from the experiment to assess mortality at each time point. The asterisk (*) indicates that treatment (**A**: dsRNA of *GFP* and *V-ATPase D,* **B**: dsRNA of *GFP* and *V-ATPase B*) means differ significantly (*P*<0.05) at a given time-point.

Separate dsRNA feeding experiments were conducted to determine nymph survival at 2-day intervals (Fig. 3A and 3B). On average, feeding nymphs dsRNA of *GFP* had no apparent effect (*P* = 0.14) on nymph survival between two and four days of exposure. There were no significant effects of dsRNA-*V-ATPase D* ingestion on nymph survival at two (*P* = 0.14) and four (*P* = 0.66) days compared to the control group (Fig. 3A), a finding consistent with the normalized abundance of this transcript during these time-points (Fig. 1). Similarly, there were no significant differences in nymph survival after *dsRNA-V-ATPase B* ingestion at 2-days (*P* = 0.37) and 4-days (*P* = 0.18) compared to those fed on dsRNA-GFP (Fig. 3B). Transcript abundance of *V-ATPase B* was reduced at the 2-day and 4-day time points but was most dramatically reduced at the later sampling time. At six days there were significant differences in survival between the dsRNA-*V-ATPase D and B* treatments and the dsRNA-*GFP* control group (*P* = 0.05, *P* = 0.04); the proportion of live nymphs fed dsRNA of *V-ATPase D* and *V-ATPase B* was significantly reduced 64% (P = 0.05) and 22% (*P* = 0.04), respectively during this time-frame.

### Mortality of nymphs injected with dsRNA of *V-ATPase B* or *D*

**Figure 4:**
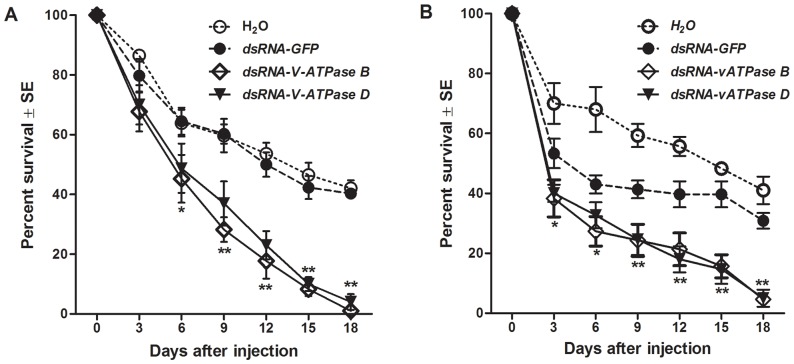
*Peregrinus maidis* mortality after injection with dsRNA. Fifth-stage nymphs were injected with 40 nL H_2_O or 40 nL (200 ng) of dsRNA (*V-ATPase B*, *V-ATPase D* or *GFP*). The experiment was performed twice, *i.e.*, two independent biological replicates (**A and B**). Each point represents the mean ± standard error of the mean of n  =  three experimental replicates with 20 insects per replicate-cage monitored repeatedly over time. The single asterisk (*) indicates that only dsRNA-V-ATPase B treatment mean differs significantly (*P*<0.05) from either control group mean; a double asterisk (**) signifies that both the means of dsRNA-V-ATPase B and D differ significantly (*P*<0.05) from that of the controls.

Survival of dsRNA-injected nymphs was determined at 3-day intervals for two control (water or dsRNA-*GFP*) and two treatment (dsRNA of *V-ATPase B* or *D*) groups. The results of two independent experiments are presented in Fig. 4A and B. In general, the two treatment groups resulted in similar reductions in the proportion of living insects over the course of the 18-day experiment (*P*>0.3), and survivorship in either V-ATPase treatment group was significantly lower than that of either control group at 9-days after injection (*P*<0.05). In comparison to the control groups, significant reductions in the proportions of surviving insects (*P*<0.05) was first documented earlier for the dsRNA-*V-ATPase B* treatment (six and three days post injection in the first and second trial, respectively) compared to the dsRNA-*V-ATPase D* treatment (nine days post injection, both trials). The survivorship in the V-ATPase treatment groups continued to decline significantly (*P*<0.05) for three of the four subsequent 3-day intervals in both trials of the experiment. By 18 days post-injection, an average of 3% to 5% of the nymphs survived treatment with dsRNA of *V-ATPase B* or *D*, while 36% to 42% of the insects survived injection with H_2_O or dsRNA of *GFP*.

Notably, there were some significant differences documented between the two trials (*P*<0.05 for experiment main effect and interaction terms involving experiment). First, there were no significant differences between the two control groups (*P*>0.40) in the first trial (Fig. 4A); however, this was not the case in the second trial (Fig. 4B), where injection with dsRNA-*GFP* resulted in significant reductions (*P*<0.02) in survival compared to the water control over the course of the experiment. Second, in the first trial, all of the control and treatment groups exhibited significant reductions in the proportion of surviving insects between three and six days post injection (*P*<0.05, Fig. 4A); while in the second trial, these reductions were documented earlier between time of injection and three days after injection (*P*<0.05, Fig. 4B). Despite the difference observed between water and GFP dsRNA for the second trial, the mortality for the GFP treatment in trial 2 did not dramatically change after the 6-day time point and was more similar to the reduction observed in trial 1. Regardless of these trial differences, the overall trend of increased mortality in dsRNA-V-ATPase-treated insects was consistent.

### Fecundity and morphology of insects injected with dsRNA of *V-ATPase B* or *D*

**Figure 5:**
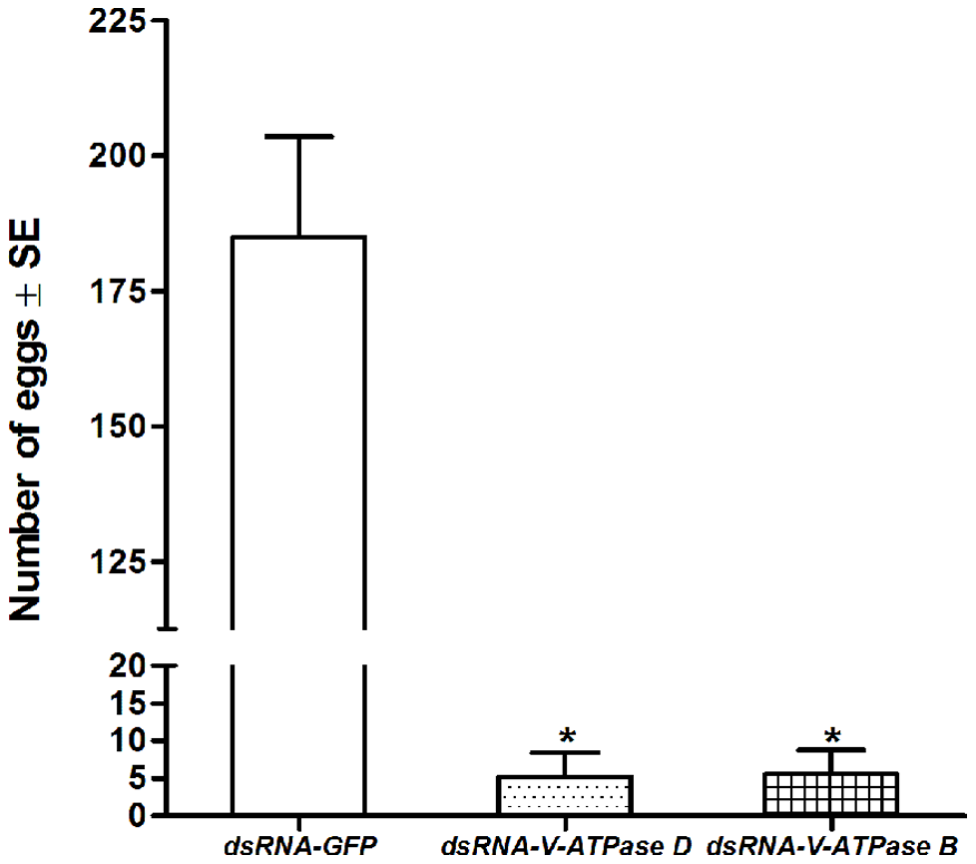
Fecundity of *Peregrinus maidis* after injection with dsRNA. Newly emerged, 24-h-old female and male planthoppers were injected with 50 nL (250 ng) dsRNA of *GFP*, *V-ATPase B* or *V-ATPase D*. Following injection, five replicate mating/oviposition cages were prepared per treatment with each cage containing two females and two males on a single corn plant. Seven days after injection, eggs laid in corn leaves were enumerated. Each bar represents the mean and standard error of the mean of n  =  two independent biological replicates of the experiment. The asterisk (*) indicates that treatment means differ significantly (*P*<0.05) from the dsRNA-GFP control group.

**Figure 6:**
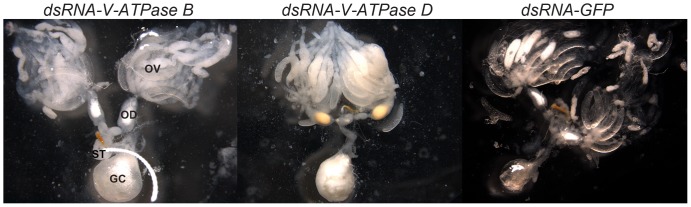
The effect of RNAi of V-ATPases on *P. maidis* female reproductive organs. (**A–C**) Young adult insects were injected with 250 ng dsRNA of *V-ATPase B, V-ATPase D,* and *GFP*. (**A and B**) The *V-ATPase B* and *V-ATPase D* dsRNA injected females have deformed ovaries full of fat tissues, and a few deformed oocytes. (**C**) Insects injected with dsRNA of *GFP* had ovaries that were full of developed oocytes. OV, ovary; OD, oviduct; ST, spermatheca; GC, genital chamber.

To determine if RNAi of *V-ATPase B* or *V-ATPase D* affects fecundity of *P. maidis*, young adult females and males (less than 24 h old) were injected with dsRNA of *GFP*, *V-ATPase B* or *V-ATPase D*, and paired on healthy plants for a week allowing them to mate and lay eggs. On average, matings between adults injected with dsRNA of either *V-ATPase B* or *D* resulted in a 97% reduction in number of eggs produced (*P*<0.0001) compared to adults injected with dsRNA of *GFP* (Fig. 5). There was no significant difference in egg numbers between the two dsRNA-V-ATPase treatments (*P* = 0.67). To further investigate the effect of V-ATPase gene knockdown, female reproductive organs were examined microscopically. Examination of female ovaries after dissection revealed that insects treated with dsRNA of *V-ATPase B* or *V-ATPase D* contained few mature oocytes, and the ovarioles appeared deformed with abnormal fat tissues (Fig. 6A and B) when compared to insects injected with dsRNA of *GFP* (Fig. 6C). The control group insects had ovaries that contained fully mature oocytes, and well organized fat tissue in ovarioles (Fig. 6C). When 5^th^-stage nymphs were injected with dsRNA of *V-ATPase B* or *V-ATPase D*, the insects did not lay any eggs at 7-days after eclosion. Examination of the insects injected as nymphs revealed that *V-ATPase B* or *V-ATPase D* dsRNA injected 5^th^-stage nymphs did not develop normal reproductive organs after eclosion compared to dsRNA-*GFP* injected insects and were similar to abnormalities observed in insects injected as adults (data not shown). These findings indicate that V-ATPase may be important for oocyte formation.

**Figure 7:**
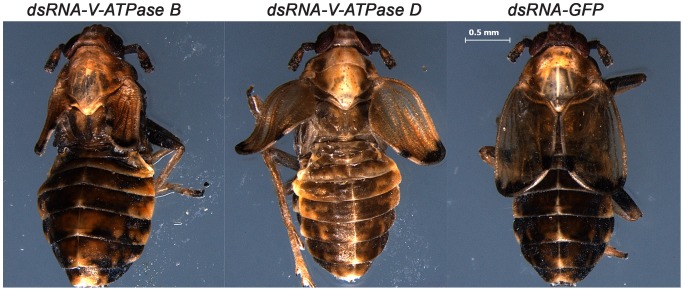
The effect of dsRNA of *V-ATPase B*, *V-ATPase D,* and *GFP* on *P. maidis* development. dsRNA (200 ng) of *V-ATPase B*, *V-ATPase D* or *GFP* was injected into 5^th^ – stage nymphs. After 10 days, all *GFP* dsRNA injected insects developed normally, but 90% and 75% of the insects injected with *V-ATPase B* and *V-ATPase D* dsRNA, respectively, were deformed. The most obvious deformity was misshapen wings, *i.e.*, curly and short.

Injection of nymphs with either dsRNA-*V-ATPase* also resulted in wing deformation. Seventy percent of nymphs injected with dsRNA of *V-ATPase B* and *D* developed deformed wing shape noted at eclosion, and the resulting females did not lay any eggs by 7-days after eclosion (Fig. 7). In contrast, 90% of the nymphs injected with *GFP* dsRNA developed normal wings and produced a large amount of eggs by 7-days after eclosion.

## Discussion

RNAi is a powerful technique for elucidating gene function in insects. In this study, we established a dsRNA delivery method in *P. maidis* to study gene function of two V-ATPase subunits. *In vitro*-synthesized dsRNA was used to feed or inject *P. maidis* nymphs and adults to investigate the relative abundance of target gene transcripts, *i.e.*, magnitude of gene knockdown. Feeding and injection of dsRNA significantly reduced the transcript levels of *V-ATPase B* and *V-ATPase D*; however, RNAi efficiency of dsRNA injection (more than 10-fold reduction) was higher than that of ingestion (approximately 2-fold reduction). Knockdown of either *V-ATPase B* or *V-ATPase D* also resulted in two observable and quantifiable phenotypes: increased mortality and reduced fecundity.

Finding a suitable dsRNA delivery method for a specific insect species of interest is a critical step for gene function studies and development of RNAi-based pest control strategies. Microinjection delivery of dsRNA into the body of insects is one of most effective methods for systemic RNAi [28] and has been widely used in many insect species, like *Blattella germanica* [29], *A. pisum* [30], *N. lugens* [16], and *Rhodnius prolixus* [31] etc. The delivery of dsRNA via microinjection can not only reduce target gene transcript levels, but also lead to phenotypic changes or even increased mortality of the tested insects. For example, injection of ecdysteroids nuclear receptor (RXR-USP) dsRNA in *B. germanica* nymphs arrested development at the end of nymph stage and they were unable to molt into adults [29]. In our study, we used microinjection for dsRNA delivery to nymphs and adults of *P. maidis*. The significant transcript reduction of *V-ATPase B* and *V-ATPase D* subunits (≥4-fold reduction) was obtained after dsRNA injection for both developmental stages. *P. maidis* injected with the dsRNA of *V-ATPase B* or *V-ATPase D* had significantly lower survival and reproductive capacities (*i.e.*, egg production) than those insects injected with dsRNA of *GFP* or H_2_O. The more rapid and effective reduction of transcript abundance by injection may be attributed to the following possible reasons: i) the amount of dsRNA delivered to the insect is greater; ii) dsRNA is delivered at one time point and the large synchronous dose may be a more effective elicitor of RNAi in *P. maidis*; and/or iii) the gut presents a barrier for dsRNA uptake that injection overcomes [32]. The delivery of dsRNA by microinjection is a very promising technique for the study gene function of *P. maidis*.

Although microinjection of dsRNA into insect body resulted in high efficiency of gene expression inhibition and led to phenotypic changes, there are some limitations to the micro-injection delivery system. There are significant side-effects of injection due to the pressure exerted on the insect during injection and the resulting wound reduces insect survival. However, measurable knockdown of genes occurred earlier through microinjection than feeding, i.e. 2-days vs. 6-days of feeding. The use of microinjection for dsRNA delivery is well-suited for functional analysis of genes in the laboratory and could be combined with delivery of pathogens. In contrast, oral delivery of dsRNA has a practical application as an insecticide in the field. Compared to other methods, dsRNA feeding is attractive primarily because it is convenient and easy to manipulate and it causes less physical damage to the insect than microinjection at time of application [15]. In our study, diet containing dsRNA of *V-ATPase D* fed to 3^rd^-stage *P. maidis* nymphs showed a significant reduction in transcript abundance after 6-days feeding and corresponded to the increase in mortality observed after 6-days feeding. Similarly, supplying dsRNA of another target gene, trehalose phosphate synthase, in artificial diet resulted in the reduction of target gene transcripts and mortality in another planthopper, *N. lugens* [15]. However, the timing of knockdown and mortality was much faster than we observed in *P. maidis* and was first observed at 2-days after feeding and persisted for up to 10 days [15]. In contrast, feeding of *V-ATPase E* dsRNA to *N. lugens* resulted in significant reductions in transcript abundance but there was no apparent effect on mortality at 2-, 6- and 10-days post-feeding [33]. The success of RNAi by diet containing dsRNA is a first step towards development of transgenic plants expressing dsRNA to control insect pests in field.

The subunits of V-ATPase have been identified as essential genes that are effective targets for RNAi [14], [33]. V-ATPase is a multi-subunit membrane protein that exploits ATP binding and hydrolysis to transport protons across membranes against a concentration gradient [19]. In *Drosophila*, 14 subunits of V-ATPase (V1 and V0 domains) are encoded by 33 genes [34]. Apart from five subunits in the V1 domain that are encoded by single genes (B, C, E, G, and H), each V-ATPase subunit is encoded by more than two genes, and as many as five genes encode the V0 subunits a and c [35]. In the *P. maidis* gut-specific cDNA library, we identified four V-ATPase subunits (B, D, E, and G) [25], and two of them (V-ATPase B and D) were chosen to document their specific functions in *P. maidis*. In *Drosophila*, V-ATPase A (*vha68-2*), V-ATPase B (*vha55*) and V-ATPase D (*vha36-1*) subunits were generally expressed in many tissues, namely ovary, testes, accessary gland, oviducts, antennal palps, midgut, hindgut and rectum and Malpighian tubules [24], [35]. The *V-ATPase A* subunit has been chosen as a target gene to knockdown through oral delivery and injection, and increased mortality was observed in several species, like *D. virgifera* [14], *Bemisia tabaci* [36], *Bactrocera dorsalis* [37] and *M. sexta* [17]. Similarly, knockout of V-ATPase genes in *Drosophila* by P-element insertion resulted in a lethal phenotype and caused an obvious phenotype of clear Malpighian tubules at embryonic or early larval stages [35]. In *P. maidis*, knockdown of *V-ATPase B* or *V-ATPase D* also led to a similar lethal phenotype (high mortality) in nymphs.

When nymphs of *P. maidis* were injected with dsRNA of either *V-ATPase D* or *V-ATPase B*, they lost the ability to deliver eggs as adult females. Microscopic examination of dissected insects revealed that female reproductive organs were underdeveloped. When newly emerged female and male adults were injected with dsRNA-*V-ATPase B* or *D*, then paired and allowed to mate, females oviposited fewer eggs than individuals injected with dsRNA-*GFP*. Similarly, deformed ovaries and oocytes were observed in females. In *Drosophila*, V-ATPase is abundant in apical and lateral follicle-cell membranes, nurse-cell membranes and the oolemma, and plays a role in follicle growth, especially in vesicle acidification and in yolk processing [38]. Brown et al. [39] found that defective V-ATPase also led to *D. melanogaster* male infertility and this may have also contributed to reduced egg production by the planthoppers. The abundance of V-ATPase in these insect tissues is consistent with our finding that knockdown results in reduced fecundity. The discovery of female reproduction organ deformity and low fecundity in *P. maidis* are two biologically-relevant phenotypes of V-ATPase knockdown. The effect of *V-ATPase D* and *V-ATPase B* subunit knockdown on *P. maidis* mortality or fecundity was similar, a finding that concurs with previous findings that *V-ATPase B* and *D* are expressed in similar drosophila tissues and the disruption of any gene encoding one subunit of V-ATPase influences the holoenzyme assembly and function [35].

In Drosophila, RNAi knockdown of V-ATPase subunits including V-ATPase B and D lead to obvious wing phenotypes [40]. Additionally, analysis of VhaPRR (a transmembrane protein that is an accessory subunit of the V-ATPase proton pump) function revealed that this protein is a modulator of canonical Wnt signaling in larval and adult wing tissue [40]. The curly wing phenotype observed in *P. maidis* is consistent with the described role of V-ATPase in Drosophila wings.

This is the first report describing RNAi in *P. maidis* by dsRNA injection and ingestion, and its success can be exploited through transgenesis and expression of dsRNA in plants to control this devastating agricultural pest in the future. In addition to insect control, RNAi will be a useful tool for exploring virus-vector interactions. *P. maidis* is a vector of two important viruses, MMV and MStV, and prior to this study there were no effective tools for examining the functions of genes associated with vector competence of *P. maidis*. With the development of RNAi and the EST resources for *P. maidis*, we can now begin to identify insect molecules involved in recognizing and responding to virus infection. Additionally, RNAi in *P. maidis* will prove to be a useful technique for investigating the function of other genes associated with MMV acquisition and transmission.
